# Leg muscle cross-sectional area measured by ultrasound is highly correlated with MRI

**DOI:** 10.1186/s13047-021-00446-y

**Published:** 2021-01-12

**Authors:** Joshua K. Sponbeck, Clint R. Frandsen, Sarah T. Ridge, Derek A. Swanson, Dallin C. Swanson, A. Wayne Johnson

**Affiliations:** Exercise Sciences Department, Brigham Young University, 1000 N University Ave, Provo, UT 84604 USA

**Keywords:** Ultrasound, Magnetic resonance imaging, Leg muscles, Cross-sectional area

## Abstract

**Background:**

The leg muscles are important for balance, posture, and movement during static and dynamic activity. Obtaining cross-sectional area measurements (CSA) of the leg muscles helps researchers understand the health and force production capability of individual leg muscles. Therefore, having an easy to use and readily available method to assess leg muscle CSA is needed. Thus, the purpose of this study was to compare the magnitude, repeatability, and validity of CSA measurements of select leg muscles from ultrasound (US) and the current gold standard, magnetic resonance imaging (MRI).

**Methods:**

20 healthy volunteers participated in this study. Each participant was imaged via US and MRI. The muscles of interest obtained on each participant consisted of the tibialis anterior at both 30 and 50% of the shank length, tibialis posterior at both 30 and 50% of the shank length, the flexor digitorum longus, the fibularis (peroneus) longus, and the fibularis (peroneus) brevis.

**Results:**

Strong Pearson correlations were seen for all of the muscles when comparing US to MRI with a range from .7840 to .9676. For all measurements, standard error of the measurement ranged from .003 to 0.260 cm^2^. Minimum detectable difference for muscle measurements ranged from .008 cm^2^ for MRI fibularis longus and fibularis brevis to .693 cm^2^ for MRI of tibialis anterior at 30%. US minimum detectable difference ranged from .125 cm^2^ for the tibialis posterior muscle at 30% to .449 cm^2^ for the tibialis anterior muscle at 50%.

**Conclusions:**

Based on these results ultrasound is a valid method to obtain CSA of muscles of the leg when compared with MRI.

## Background

Imaging and analysis of muscle cross-sectional area (CSA) can give understanding of the health [1] and force production potential of a muscle [2]. This can be particularly useful for assessment of muscles that are hard to isolate during functional testing, for example in the lower leg (knee to ankle, anatomically known as the leg), where several muscles perform the same actions. As there are no commercially readily available devices used to assess strength of specific or isolated leg muscles, anatomical muscle CSA provides the ability to infer force production of these muscles [2].

There are currently limited ways to assess muscle CSA in vivo. These include magnetic resonance imaging (MRI), computed tomography, and ultrasound imaging (US). MRI has been validated and is now largely considered the “gold standard” for comparison of other imaging methods, however MRI is expensive, time consuming, and not always readily available [3–6]. Computed tomography has limited availability for these purposes in the research and clinical settings as a result of the consequences of repeated radiation exposure, as well as cost [7]. Ultrasound imaging is a relatively low-cost alternative that is becoming readily available in the research and clinical settings [8]; however, validation of US compared to MRI is necessary for specific muscle groups.

The use of US imaging has several advantages for the evaluation of soft tissue. It allows for reliable, high-resolution assessment of soft-tissue under static and dynamic conditions [9–11]. Dynamic movement patterns, such as muscle contraction, can be recorded in retrospective video clips (Cine-loops), that have been shown to decrease operator imaging and measurement error [11]. As with other imaging modalities, however, US imaging is operator dependent, requires significant operator training, and has a limited field of view that requires detailed anatomical knowledge of the imaged area [8].

While US measurements of several muscle groups have been validated with MRI, few studies have reported this data from any leg muscles [3, 8, 12, 13]. The specific arrangement and anatomical relationships of leg muscles present unique challenges to image acquisition and measurement [14]. As these leg muscles are crucial during dynamic movement [15] as well as during static posture and balance [16], the ability to assess these muscles’ CSA accurately, reliably, and quickly is necessary.

Therefore, the primary purpose of this study was to compare the magnitude, repeatability, and validity leg muscle CSA measurements acquired from US images compared with images taken via MRI. We hypothesized that US imaging and subsequent CSA analysis of selected muscles of the leg would correlate closely with those same muscles analyzed using MRI.

## Methods

10 males and 10 females completed this study (mean and (SD), age = 34.15 (16.55) years, weight = 80 (4.88) kg, height = 169.72 (34.78) cm). All participants were volunteers, ages 18 years or older, who did not have an extremity injury within the previous one month or leg/foot surgery within the previous year. Each participant read and signed an informed consent approved by the University’s Institutional Review Board (study protocol, IRB2019–375). Study participants reported for two visits that consisted of the US session, and the MRI session. On average the two visits were 10 days apart for study participants.

In order to ensure consistency of measurement of each muscle, the linear distances from the lateral knee joint line to the inferior point of the lateral malleolus, as well as the linear distance from the medial knee joint line to the inferior point of the medial malleolus were measured. From these measurements, the 30 and 50% distances from the knee joint line were determined and marked with a soft-tipped marker. These measurement locations were recorded and used in both MRI and US sessions.

### MRI protocol

Prior to entering the MRI machine, participants completed a safety screening, in the waiting room of the MRI facility. Upon completion, fish oil tablets attached to a Velcro strap were placed at the previously measured markings of 30 and 50% of the shank length. The fish oil tablets allowed the researchers to consistently locate the appropriate slices to measure at the correct location of the shank. A 3 Tesla magnet (TIM-Trio 3.0 T MRI, Siemens, Erlangen, Germany) was used to scan the left leg first, then the right leg. 30% shank length images were obtained first, followed by the 50% shank length image for each leg. Participants were lying supine and placed feet first into the magnet. The initial localizer scan was centered on the marked location being imaged. T1 weighted MRI images were acquired using a Siemens sequence using an axial orientation, and an acquisition time of 20 s. The resolution was 1.56 mm by 1.95 mm with a slice thickness of 6 mm and a space between slices of 3 mm. The resolution matrix was 256 × 205. An 8-channel knee coil was used to obtain a total of 10 images at each location. Repetition Time (TR)=7.3 ms and Echo Time (TE)=3.6 ms.

**Fig. 1:**
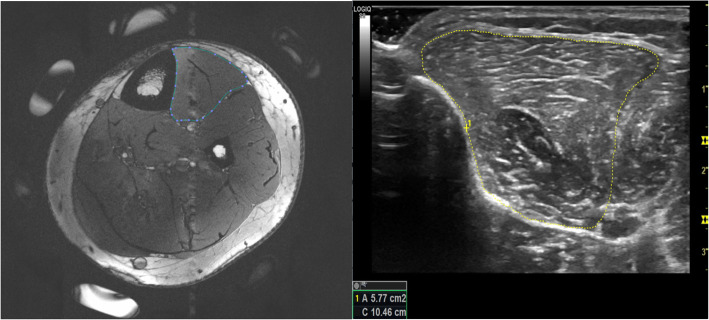
MRI (left) and US (right) images of the tibialis anterior muscle with associated segmentation of the muscle

All images obtained from the MRI scans were loaded into Osirix (Pixmeo, Geneva, Switzerland) in order to obtain CSA measurements. Two CSA measurements were taken from adjacent slices of the same scan at the location of the fish oil tablets on the MRI.. Measurements were obtained by two researchers (JS and DaS) for each the tibialis anterior, the tibialis posterior, the flexor digitorum longus, the fibularis (peroneus) longus, and the fibularis (peroneus) brevis muscles. Muscles were outlined inside of the muscle fascia (Fig. 1).

### Ultrasound imaging

A researcher with 10 years of musculoskeletal US imaging experience gathered images obtained in this study. Participants sat in a relaxed position on a treatment table with an upright, inclined back and had their thigh supported by a bolster so that their calf was uncompressed. The same muscles were imaged via US (LOGIQ S8; GE Healthcare, Chicago, IL) using an ML6–15-D matrix linear transducer. Frequencies ranged between 8 and 12 MHz as determined by the scanner to enhance image clarity. Adjustments to depth, frequency, focal position, and time-gain-compensation were performed as needed to enhance the clarity of the image. Additionally, the Virtual Convex setting was occasionally used to enhance the field of view during scanning.

Short axis images of each muscle starting with the tibialis anterior were obtained at 30 and 50% of the shank length with the lateral border of the tibia serving as an anatomical landmark during imaging. The tibialis posterior was imaged at both the 30 and 50% marks of the shank from the anterior side [10]. The tibialis anterior muscle and interosseous membrane served as anatomical landmarks during imaging. The flexor digitorum longus was imaged at 50% of the shank length on the medial side just posterior to the tibia, with the tibia serving as an anatomical landmark to aid in imaging consistency. The fibularis longus was imaged at 30% of the shank length with the fibula serving as an anatomical landmark for consistency just deep to the fibularis longus. The fibularis brevis was measured at 50% of the shank length with the fibula serving as an anatomical landmark just deep to the fibularis brevis. During imaging, participants were asked to perform muscle contractions causing the imaged muscle to contract and then return to rest. Cine loops were recorded of the contraction cycle to help visualize the fascial borders of the muscles and the conformational changes within the muscle. This allowed the muscles of interest to be distinguished from the adjacent muscles and other leg structures. Two separate recordings of the contraction cycle were taken of each muscle. The transducer was removed from the leg between recordings.

CSA measurements were obtained post imaging session, from a single frame within each of the two separate cine loops.. CSA measurements were obtained using internal software on the LOGIQ S8 machine. Measurements were performed by two members of the research team (DaS and DeS) who have been previously trained to obtain CSA measurements from these specific muscles. All muscles were traced within the facial borders during rest. During US imaging Cine-loops were obtained to aid the researchers performing the measuring by viewing the muscle contraction. This analysis was similar to previously established methods [10, 11] (Fig. 1).

### Statistical analysis

Intraclass-correlation coefficients (ICC3,k) were performed to establish reliability using a CSA measurement from each MRI and US image. As we were interested in our segmentation repeatability, we chose the ICC model with fixed raters and random subjects assessed for absolute agreement. Additionally, the averages of the two measured CSAs for both MRI and US images were calculated. The averages were used in a Pearson product moment correlation to determine the validity of the US estimates of muscles’ CSA compared to the MRI. Our interpretation of Pearson’s Correlation coefficients will be based upon previous research as follows: 0.9 to 1 is very strong, 0.70 to .89 is strong, 0.5 to 0.69 is moderate, .30 to .49 is low, and 0.0 to 0.29 is negligible [17]. T-tests were performed to determine if any muscle CSA differed significantly between US and MRI and to conclude that a Bland-Altman plot analysis would be appropriate. Bland-Altman plot analysis was used to understand potential systematic error between US and MRI. To represent error inherent to each imaging modality, we calculated the standard error of the measurement (SEM) and minimum detectable difference (MDD) for both MRI and US for each of the muscles imaged using the following equations:

$$ {\displaystyle \begin{array}{c}\mathrm{SEM}=\mathrm{SD}\ \left(\mathrm{Sq}\ \mathrm{rt}\ 1-{\mathrm{r}}_{\mathrm{ICC}}\right)\\ {}95\%\mathrm{CI}\ \mathrm{SEM}=\mathrm{muscle}\ \mathrm{mean}\pm \left(1.96\ast \mathrm{SEM}\right)\\ {}\mathrm{MDD}=\mathrm{SEM}\ast 1.96\ast \mathrm{sqrt}\ 2\end{array}} $$

Statistical analyses were performed using Statistical Analysis Software (JMP pro Version 14.2, SAS Institute, Inc. Cary, NC, USA) for all statistics except for ICC_3,k_ values, which were obtained using SPSS version 26.0 statistical software (IBM Corporation, Armonk, NY). An alpha of 0.05 was used to determine significance of statistical tests.

## Results

**Fig. 2:**
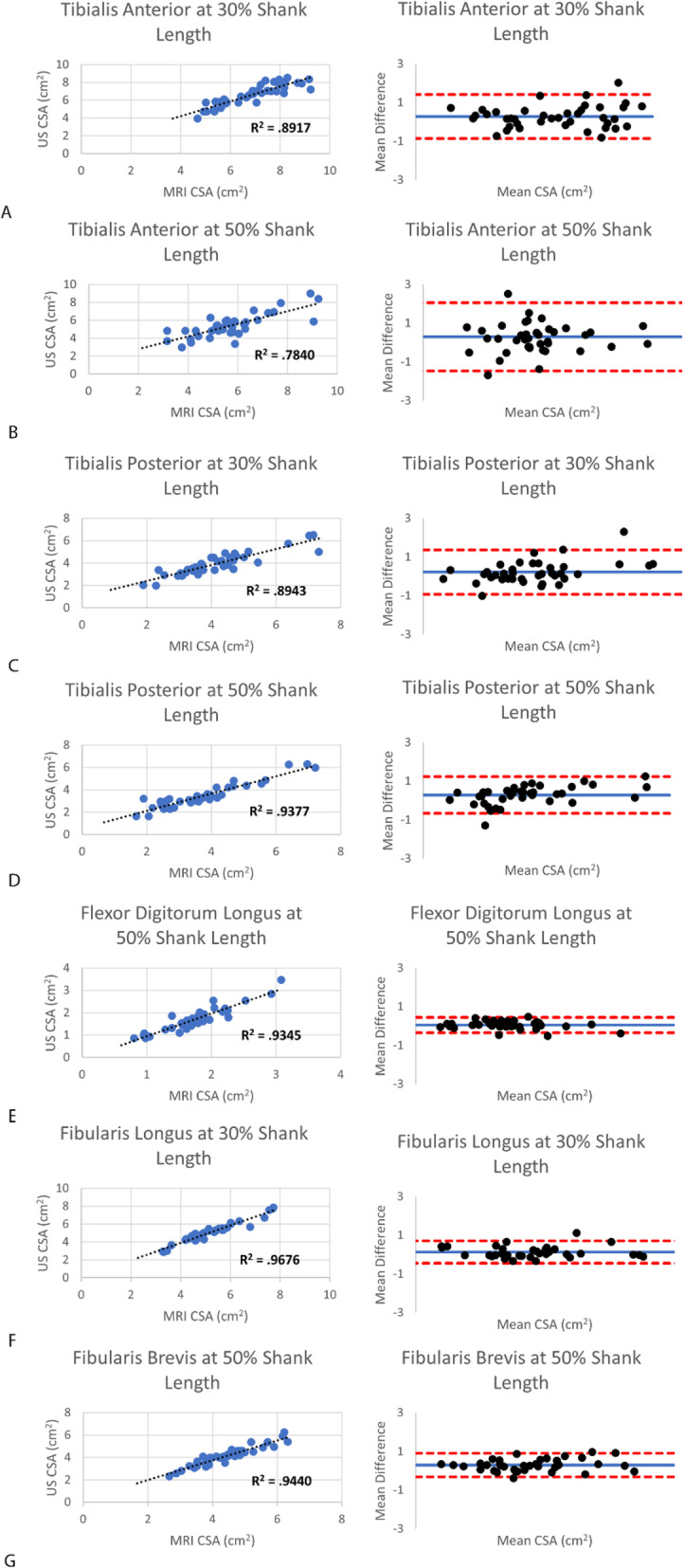
(A-F) Correlation data including R^2^ values, and corresponding Bland-Altman plots for all muscles of interest in the leg. The dotted black line in the correlation plots is the line of best fit for the data. In the Bland Altman plots the blue line represents the bias, and the red lines represent upper and lower limits of agreement (LoA = Bias ± 1.96*SD)

Table 1 contains all assessed mean muscle CSA values for US and MRI measurements, ICC values, SEM, and MDD. The correlations between MRI and US imaging and segmentation were strong to very strong with a range from 0.784 to 0.968 (Fig. 2) [17]. No muscle CSA means were significantly different between US and MRI measures of CSA with *p* values ranging from 0.164 to 0.990. Average CSA measured from MRI were slightly larger for all of the muscles (except for fibularis longus, which was nearly equal), as illustrated by the positive biases on the Bland-Altman plots (Fig. 2, Table 2). For all measurements, SEM ranged from 0.003 to 0.260 cm^2^. MDD for muscle measurements for both US and MRI ranged from 0.008 to 0.693 cm^2^. Individual reliability was excellent for both MRI and US for each muscle ranging from 0.958 to 0.999.

**Table 1:** Comparison of muscle CSA by MRI and US. Mean (SD), ICC, and SEM with confidence intervals for muscle CSA

	US CSA (SD), cm^**2**^	US ICC (95% CI)	US SEM (95% CI for muscle size), cm^**2**^	Minimum Detectable Difference, cm^**2**^	MRI CSA (SD), cm^**2**^	MRI ICC (95% CI)	MRI SEM (95% CI for muscle size), cm^**2**^	Minimum Detectable Difference, cm^**2**^
**Tibialis Anterior** **30%**	6.60 (1.20)	0.996 (0.992, 0.998)	0.085 (6.43, 6.77)	0.236	6.88 (1.28)	0.963 (0.930,0.980)	0.250 (6.39, 7.37)	0.693
**Tibialis Anterior 50%**	5.39 (1.28)	0.985 (0.973, 0.992)	0.162 (5.07, 5.71)	0.449	5.69 (1.42)	0.991 (0.982,0.995)	0.135 (5.43, 5.95)	0.374
**Tibialis Posterior 30%**	3.96 (1.01)	0.998 (0.996, 0.999)	0.045 (3.87, 4.05)	0.125	4.18 (1.27)	0.958 (0.921, 0.978)	0.260 (3.67, 4.69)	0.721
**Tibialis Posterior 50%**	3.44 (1.10)	0.997 (0.995, 0.997)	0.060 (3.32, 3.56)	0.166	3.72 (1.33)	0.999 (0.999, 1.00)	0.013 (3.69, 3.74)	0.036
**Flexor Digitorum Longus**	1.66 (.569)	0.990 (0.983, 0.995)	0.054 (1.55, 1.77)	0.150	1.71 (.52)	0.995 (0.990, 0.997)	0.044 (1.62, 1.80)	0.122
**Fibularis Longus**	5.26 (1.25)	0.996 (0.993, 0.998)	0.073 (5.11, 5.40)	0.202	5.26 (1.14)	0.998 (0.995,0.999)	0.003 (5.25, 5.27)	0.008
**Fibularis Brevis**	4.18 (.973)	0.993 (0.987, 0.996)	0.074 (4.03, 4.33)	0.205	4.38 (.942)	0.997 (0.994,0.998)	0.003 (4.37, 4.39)	0.008

**Table 2:** Bias, lower limit of agreement (LoA), and upper LoA for each muscle imaged in the leg

	Tibialis Anterior 30%	Tibialis Anterior 50%	Tibialis Posterior 30%	Tibialis Posterior 50%	Flexor Digitorum Longus	Fibularis Longus	Fibularis Brevis
Bias	0.27	0.30	0.22	0.28	0.04	0.13	0.30
Lower LoA	−0.87	−1.46	−0.92	−0.66	− 0.36	−0.45	− 0.31
Upper LoA	1.42	2.06	1.36	1.23	0.44	0.70	0.90

## Discussion

The primary purpose of this study was to establish the validity of leg muscle CSA measurements acquired from US images. The magnitude and measurement repeatability of muscle CSAs obtained from US imaging were compared with those obtained from MRI. The results of our study indicate that US imaging and subsequent segmentation of leg muscles are strongly to very strongly correlated with MRI. We also found excellent intra-rater repeatability for both US and MRI.

Our results support previous research showing muscle CSA when imaged with US is valid and correlated with MRI. Muscle groups that have been previously used to validate and correlate US with MRI include the quadriceps muscles [18, 19], rotator cuff muscles [13], hamstring muscles [20], biceps and triceps brachii muscles [21], and core trunk muscles [8, 22]. Our values fall within the upper range of correlations reported in these studies, and range from 0.53 to 0.99. No studies have reported a comparison of leg muscle CSA between US and MRI, though a single study reported very strong correlation of muscle volume measurements of the tibialis anterior muscle between these imaging modalities [12].

Although not statistically different from MRI, average US muscle measurements were slightly smaller for nearly all muscles measured. Ahtianinen et al. displayed similar findings, with smaller rectus femoris average muscle US CSA when compared to MRI during a training study [18]. Possible explanations for smaller US means include differences in processing algorithms between US and MRI, measuring planes between imaging modalities, or US probe compression of muscle. However, other researchers dispute these possible reasons [23]. Despite these differences, previous intervention studies have shown that changes in muscle size are consistent when measured with US and MRI [18]. These data suggest that either imaging modality can be used to track changes over time.

When using US as an imaging modality, the operator dependence is important to take into account. US offers a limited field of view, is sensitive to operator technique, and requires anatomical knowledge of the imaged area. To address this potential limitation, when multiple clinicians and/or researchers work together, they should practice similar techniques and assess reliability. For example, the use of cine loops has been shown to increase inter-rater reliability [11]. While less operator dependent, MRI is still highly sensitive to participant positioning [24]. Regardless of imaging modality used, it is important to calculate measures such as the MDD when tracking muscle size changes over time. Small MDD provide confidence that true changes occurred, as opposed to error induced by the operator. The repeatability across operators and measures deserves further refining and research. The current study reported similar US leg muscle CSA values compared to previous US research when available. Among direct comparisons of the flexor digitorum longus muscle there was 99% similarity in muscle CSA average with previous research performed by our group [11]. The fibularis brevis muscle had an average mean muscle CSA of 4.18 cm^2^ as measured by US, which is similar to previous research that measured 4.09 cm^2^ (9) and 3.5 (24) cm^2^. Other muscles from the current study were difficult to compare to previous studies. While our research group has previously demonstrated US measurement and segmentation of the tibialis posterior muscle, to our knowledge this is not being performed elsewhere [10]. The fibularis longus and tibialis anterior muscle sizes have been imaged at different locations of the muscle [9, 25, 26] in previous studies, or segmented and measured using width or volume only [11, 12] and not CSA as was used in this current study.

US may provide several advantages to clinicians and researchers for obtaining muscle CSA values, as opposed to MRI. Possible benefits of US may include decreased imaging time, imaging safety, reduced cost, modality availability, visualization of muscle contraction, and potential use to provide biofeedback. During the current study the US imaging session lasted 15 min including participant preparation time, compared with 30 min for the MRI session including participant preparation time. US has virtually no contraindications and very limited side effects making imaging possible to those who may not be indicated for an MRI, such as those with metal implants or a pacemaker [24]. The US unit may be much more readily available, and a fraction of the cost [8]. Additionally, as US measurements are performed in real time, they may be used by clinicians to provide biofeedback for patients. The use of biofeedback has resulted in improved performance and long-term contractile ability of a muscle [22].

### Limitations

One consideration when comparing measurements from different imaging modalities is the amount of day-to-day variability in muscle CSA. Each participant was imaged with both US and MRI one time. Previous research has shown slight muscle CSA variations (ranging from 1 to 4.7%) across days [23]. Although our participants were imaged on different days, participants were imaged at similar times of the day, and physical activity was controlled in an attempt to limit variability.

## Conclusion

US is a reliable and valid method of measuring muscle CSA for the tibialis anterior, tibialis posterior, flexor digitorum longus, fibularis longus, and fibularis brevis muscles when compared with MRI. Being able to use US rather than MRI may help researchers and clinicians spend less time completing participant imaging and data analysis, increasing efficiency and lowering cost. Additionally, US allows for dynamic testing and biofeedback. For some researchers and clinicians US is also a more readily available modality and therefore is an important tool when desiring to view and analyze individual muscle CSA of the leg.

## Data Availability

The datasets used and/or analyzed during the current study are available from the corresponding author on reasonable request.

## Abbreviations

CSA: cross-sectional area
MRI: magnetic resonance image
US: Ultrasound
